# Radiation-Induced Low-Grade Glioma following Radiotherapy for Squamous Cell Carcinoma of the Scalp: Case Report and Literature Review

**DOI:** 10.1155/2024/1907435

**Published:** 2024-07-09

**Authors:** Moayad. M. Z. Ahmed, Fawaz. E. M. Abdelradi, Rabee A. ELfeel

**Affiliations:** ^1^ Aliaa Specialist Hospital, Omdurman, Sudan; ^2^ Bahri Teaching Hospital, Khartoum North, Sudan; ^3^ Doctor Specialized Hospital Bahri Teaching Hospital, Khartoum North, Sudan

## Abstract

**Introduction:**

Radiation-induced gliomas (RIGs) were reported in the literature in general. In most of the reported cases and the reviewed articles, patients have a history of primary intracranial tumors like craniopharyngioma, medulloblastoma, and ependymoma, and the commonly resulting secondary tumors are meningiomas and sarcomas, mainly not gliomas. *Case Presentation*. A 50-year-old woman had a history of left scalp temporal region periauricular squamous cell carcinoma, which was verified by the histology result of a biopsy 11 years ago. On the basis of that, she began receiving low-dose radiation sessions when she was 39 years old. She exhibits cranial symptoms and a radiological sign of cancer 9 years later. After a successful excision procedure, histology revealed diffuse astrocytoma Grade 2. Our case is suspected to fit the criteria for being identified as RIG, which is a syndrome that is thought to occur infrequently in the literature.

**Conclusion:**

In conclusion, the way that this condition manifested in our case is considered rare due to old age and the low doses of radiation received. Despite being an important part to confirm the diagnosis, genetic studies were unfortunately not done in our case, but we mainly based on the criteria mentioned by Cahan et al., which are mainly taken from the clinical history and histopathology. Here, we present an example of considering such a diagnosis when suspected clinically, but a genetic study for confirmation should be thought of even if it is not available in the locality.

## 1. Introduction

Radiation is used as a therapeutic tool in the management of tumors, especially those of the central nervous system, and is a known practice in the history of medicine. Radiotherapy itself is reported to have side effects like radiation necrosis, microangiopathy, and progressive leukoencephalopathy. Recently, the literature mentioned radiation-induced tumors, including sarcomas, gliomas, lymphomas, and carcinomas of the thyroid [1]. In the neurooncology literature, they reported what is called radiation-induced glioma (RIG), which can be defined as the tumor occurring in the field of previous radiation therapy, being histologically distinct from the condition for which the radiation was prescribed, having a sufficient latency period between the completion of the radiation therapy and the onset of the tumor, and excluding underlying conditions that predispose patients to the development of multiple tumors such as immunodeficiency syndromes, Von Recklinghausen syndrome, and xeroderma pigmentosum [2]. History-wise, RIGs were mentioned in 1953 with the development of a meningioma after treatment of an orbital astrocytic tumor in an Australian pediatric patient [3].

The increased risk of RIGs is well described in both the pediatric and adult populations and after the use of both therapeutic and diagnostic radiation. The incidence of RIGs is estimated at 0.5%–2.7%, with a latent period of approximately 15 years [1].

What makes the presented case rare is that it occurs due to therapeutic radiation just underneath the radiation field, bearing in mind the latency period between exposure and the glioma diagnosis. Extensive retrospective cohort data in pediatric populations after therapeutic intracranial radiation show a clearly increased risk of glioma incidence that is both patient age and radiation dose and volume-dependent. Children (especially those under 5 years of age) are at greater risk for RIGs than adults.

Data on adults is more limited but shows an increase in risk in certain exposed groups. In both populations, there is no evidence linking increased risk to routine exposure to diagnostic radiation [4]. The literature mentions that patients with RIGs have a significantly poorer prognosis without reradiation [4].

## 2. Focused Case Scenario

**Figure 1:**
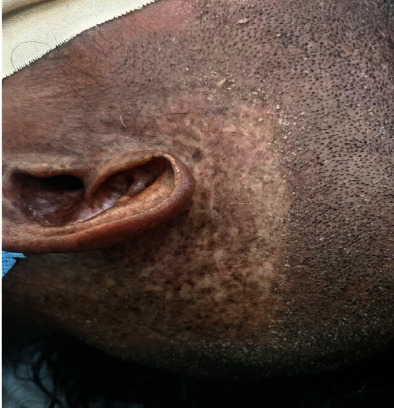
Photo of the left ear and scalp area showing scarring denoting the stigmata of the skin carcinoma with the effect of previously focused radiotherapy.

A lady with no medical history of comorbidity (HTN or DM) or history of known chronic illness was diagnosed with scalp skin squamous cell carcinoma on the left periauricular area (moderately differentiated) (Figure 1) at the age of 39 years in 2007. Her record of radiotherapy showed that she was taking 15 McV of fraction 10, dose/fraction: 400 cGy/fraction, field size: 6 × 7 cm^3^, application: 10 × 7 cm^3^, output factor: 0.985 cGy/MU, application factor: 1.0.

**Figure 2:**
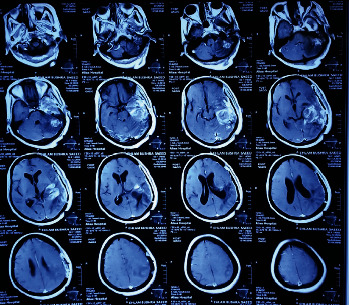
MRI brain axial cut T1-weighted image with contrast shows left temporal heterogeneously enhancing lesion featuring malignancy.

In 2016, at the age of 50, she was presented with headaches and convulsions for about 8 years after being suspected of having tuberculoma of the brain based on the radiological features. She was receiving treatment for that but had no significant response. Preoperative MRI brain with contrast (Figure 2) showed radiological features favoring malignancy rather than tuberculoma. Based on that suspicion, the patient underwent surgery with gross total resection of the lesion; the histopathology result showed a neoplasm composed of spindle cells with moderate pleomorphism and focal gemisytocytic astrocytes noted. The stroma is vascular and shows change; all features are consistent with diffuse astrocytoma grade. Postoperatively, the patient was sent to an oncologist who advised that there was no radio-chemotherapy management, but there would be serial clinical and radiological follow-ups. The patient did well in the postoperative period with significant clinical improvement and is still in long-term follow-up with the unit.

## 3. Discussion

RIGs have been mentioned and reported a lot in the literature in general. Most of the reported cases and the reviewed articles with meta-analysis revealed it as a common event seen in younger patients with a history of primary tumors commonly found to be intracranial tumors like craniopharyngioma, medulloblastoma, and ependymoma, and the commonly resulting secondary tumors are not gliomas but meningiomas and sarcomas instead [5]. Our case is seen following radiotherapy for an extracranial primary malignancy, which is squamous cell carcinoma, which is purely reported as a primary lesion. The agreed criteria for the diagnosis of RIG that are mentioned in the literature are: a secondary tumor must occur within the irradiated field; a sufficient latency period must exist between irradiation and tumor incidence; the radiation-induced tumor must be proven to be of a different histological type from that of the original neoplasm; and the patient must not have any pathologies favoring the development of tumors such as von Recklinghausen disease, Li-Fraumeni disease, tuberous sclerosis, xeroderma pigmentosum, retinoblastoma, or neurofibromatosis [6]. Our case fulfilled these criteria because it occurred on the same side of radiotherapy with different histology between the primary and secondary tumors, the latency period was about 9 years, and our patient was not known to have any previous disease considered a risk factor for developing malignancy. RIG is commonly seen in young people and frequently located in the cerebellum and spinal cord [7], while our case is seen in adulthood with a supratentorial site in the temporal lobe. RIG is mentioned in the literature as being radiation dose- or volume-dependent [4]. Of course, most cases are seen with high doses, but low doses are also found to be associated with secondary malignancy [8]. In a comprehensive review with meta-analysis about RIGs, the average radiation dose delivered to the primary lesion was 37.6 ± 20.0 Gy [9], because approximately 10% of cases developed secondary gliomas following radiotherapy treatment with less than 16 Gy. Our case was treated with 15 Gy, as mentioned in her record. Literature also talked about the pathogenesis of such events and mentioned that activation of oncogenes or inactivation of tumor suppressor genes via DNA strand breaks has been hypothesized as the main mechanism driving the development of secondary tumors after radiotherapy [10]. Somatic p53 gene mutations have been identified in radiation-induced tumors [11]. RIG is difficult to treat; radiotherapy is not always a therapeutic option as the patient may have already had prior exposure [9]. Mayer and Sminia found that reirradiated normal brain tissue could tolerate a cumulative total dose of more than 100 Gy using conventional fractionation. These observations indicate that therapeutic approaches using methods such as reirradiation might allow for prolonged disease control in some patients with RIGs [12]. A high frequency of TP53 mutations, CDK4 amplification, or CDKN2A homozygous deletion, and amplifications or rearrangements involving receptor tyrosine kinase and Ras-Raf-MAP kinase pathway genes, including PDGFRA, MET, BRAF, and RRAS2 were found in a study on postradiation glioma to determine the molecular pathogenesis. IDH1, IDH2, H3F3A, HIST1H3B, HIST1H3C, TERT (including promoter area), and PTEN, which genetically identify the primary subtypes of diffuse gliomas in children and adults, were not altered in any tumors. Less than three somatic single nucleotide variations or minor indels per mb were found in any of the gliomas. When compared to naturally occurring high-grade gliomas, the 10 high-grade gliomas showed clearly aneuploid genomes, a considerable increase in the number of intrachromosomal copy number breakpoints, and focal amplification/homozygous deletions [13].

## 4. Conclusion

Although it is an uncommon occurrence, CNS cancer after radiotherapy should be taken into consideration with long-term follow-up to detect it, particularly in highly suspected groups (high doses received and the pediatric population). To fully comprehend the pathophysiology of such a complication, a molecular analysis of the problem is essential, which was not available at that time.

## Data Availability

Data will be available from the first author upon request.
